# On the Acoustic Filtering of the Pipe and Sensor in a Buried Plastic Water Pipe and its Effect on Leak Detection: An Experimental Investigation

**DOI:** 10.3390/s140305595

**Published:** 2014-03-20

**Authors:** Fabrício Almeida, Michael Brennan, Phillip Joseph, Stuart Whitfield, Simon Dray, Amarildo Paschoalini

**Affiliations:** 1 Department of Mechanical Engineering, UNESP – FEIS, Av. Brasil, 56, 15385-000, Ilha Solteira, Brazil; E-Mails: mjbrennan0@btinternet.com (M.B.); tabone@dem.feis.unesp.br (A.P.); 2 Institute of Sound and Vibration Research—ISVR, University Road, SO17 1BJ, Southampton, UK; E-Mail: pfj@soton.ac.uk; 3 South Staffs Water plc, Green Lane, Walsall WS2 7PD, UK; E-Mail: stuartwhitfield@south-staffs-water.co.uk; 4 Hydrosave Ltd, Swallow Court, Venture Park, Kettering NN15 6XX, UK; E-Mail: sdray@hydrosave.co.uk

**Keywords:** leak detection, plastic water distribution pipes, acoustic methods

## Abstract

Acoustic techniques have been used for many years to find and locate leaks in buried water distribution systems. Hydrophones and accelerometers are typically used as sensors. Although geophones could be used as well, they are not generally used for leak detection. A simple acoustic model of the pipe and the sensors has been proposed previously by some of the authors of this paper, and their model was used to explain some of the features observed in measurements. However, simultaneous measurements of a leak using all three sensor-types in controlled conditions for plastic pipes has not been reported to-date and hence they have not yet been compared directly. This paper fills that gap in knowledge. A set of measurements was made on a bespoke buried plastic water distribution pipe test rig to validate the previously reported analytical model. There is qualitative agreement between the experimental results and the model predictions in terms of the differing filtering properties of the pipe-sensor systems. A quality measure for the data is also presented, which is the ratio of the bandwidth over which the analysis is carried out divided by the centre frequency of this bandwidth. Based on this metric, the accelerometer was found to be the best sensor to use for the test rig described in this paper. However, for a system in which the distance between the sensors is large or the attenuation factor of the system is high, then it would be advantageous to use hydrophones, even though they are invasive sensors.

## 1. Introduction

Water distributions systems are susceptible to leakage, which results in a substantial wastage of water. The social and environmental effects due to leakage are also a matter of concern. For example, up to 4 million holes are dug in the UK each year in order to install or repair buried service pipes and cables. Recently, a survey on the costs of this installation/repair work estimated that street works cost about £7 bn in losses for the UK government income annually; £5.5 bn are due to social costs and £1.5 bn is due to damage [1].

Acoustic techniques have been used for many years in the water industry to detect leaks [2], and more recently they have been applied to locate underground pipes [3] and blockages (sediment depositions) in pipe networks [4]. Correlation techniques have been in common use for water leak detection over the last 30 years [5]. In general, these techniques work well in metal pipes, but their effectiveness in plastic pipes is limited [6]. Thus, the specific problem of detecting leaks in plastic pipes using acoustics has recently been receiving increasing attention by the research community. There are two fundamental issues that affect leak detection in plastic pipes: the first is that there is considerably more uncertainty in the noise propagation speed for plastic pipes (which needs to be known *a priori* for acoustic methods to be effective); and the second, which is more important, is that leak noise does not propagate as far in plastic pipes as it does in metal pipes [7]. Hunaidi and Chu [8] have described the frequency content present in leak signals measured on a bespoke buried plastic pipe rig located in Canada. Gao *et al.* [9] have also used the data collected from this rig to gain physical insight into the problems by comparing experimental results with predictions from simple models of the correlation function in plastic pipes due to leaks.

Although there is a body of work in the literature on leak detection using acoustic methods in plastic water distribution pipes, for example [5–14], apart from [11], there is no work in which there is a direct comparison between the effectiveness of correlation for leak detection using measurements of acoustic pressure, velocity or acceleration. Reference [11] describes a theoretical study on the different types of sensors and how they combine with the pipe to act as a filter of the leak noise. The aim of this paper is to validate these findings by carrying out an experimental study in a bespoke test rig in which simultaneous measurements using hydrophones (acoustic pressure), geophones (velocity) and accelerometers (acceleration) were made. Moreover, a quality measure for the data is proposed and tested experimentally as a metric of the prominence of the peak in the cross-correlation function related to the leak noise. Two sets of data are presented, one for a strong leak where there was good signal to noise ratio, and one for a weak leak where this was not the case.

The paper is organised as follows: in Section 2 an overview of the way in which a leak is located using acoustic techniques is given. In Section 3, the dynamic effects of the pipe and the sensors on the measured leak noise are described. Section 4 is devoted to the experimental work and the processing of the data, while Section 5 discusses the results. Conclusions from this work are summarised in Section 6.

## 2. Overview of Leak Detection using Acoustic/Vibration Signals

**Figure 1.**
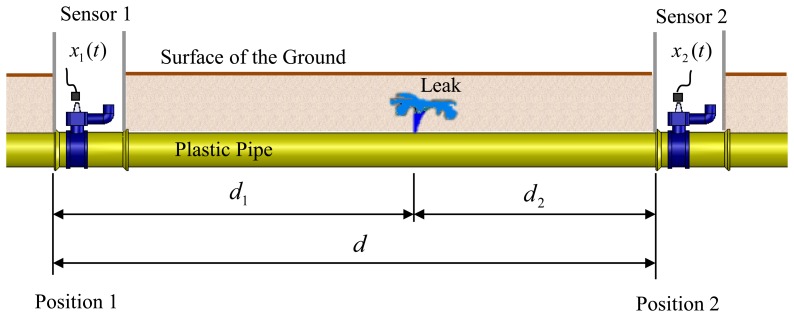
Schematic of buried water pipe in which sensors are positioned at access points either side of a leak.

Figure 1 depicts a situation typically encountered in leak detection, in which a leak occurs at an unknown position in a buried water pipe.

The leak generates broadband noise, which propagates along the pipe, both in the fluid and along the pipe-wall either side of the leak, to sensors that are located at convenient access points. These are often fire hydrants. In plastic water pipes the pipe-wall and water are strongly coupled in an acoustical sense [6,7]. This means that measurements of leak noise can, in principle, be made on the pipe or associated fittings (velocity and acceleration using geophones or accelerometers), or in the water directly (acoustic pressure using hydrophones). The difference in the arrival times of the noise at the sensors (time delay) is used to determine the position of the leak and the distance of the leak from the right-hand sensor can then be determined from [9]:

(1) $$d_{2}=\frac{d-cT_{0}}{2}$$

where *c* is the speed of propagation of the leak noise, *d* is the total distance between the sensors, and *T*_0_=(*d*_1_ − *d*_2_)/*c* is the time delay estimate. In many cases the wavespeed is estimated from tables, but it can be directly measured in-situ [12]. Note that it is often extremely difficult to obtain an accurate estimate of the propagation of leak noise from measurements, as the estimate is highly dependent upon the signal to noise ratio. This has a profound influence on the bandwidth over which there is useful data and this has a consequent effect on the estimate of *c* [12].

Setting the means of the two measured signals *x*_1_(*t*) and *x*_2_(*t*) to zero, the cross-correlation function is given by [15]:

(2) $$R_{x_{1}x_{2}}\left(\tau \right)=\text{E}\left[x_{1}\left(t\right)x_{2}\left(t+\tau \right)\right]$$

where *τ* is time delay and E[ ] is the expectation operator. The value of *τ* that corresponds to the peak in in the cross-correlation function provides an estimate of the time delay *T*_0_. It is preferable to express the cross-correlation function in a normalized form, which has a scale of −1 to +1. This is called the cross-correlation coefficient and is given by:

(3) $$\rho (\tau )=\frac{R_{x_{1}x_{2}}(\tau )}{\sqrt{R_{x_{1}x_{1}}(0)R_{x_{2}x_{2}}(0)}}$$

where *R_x_*_1_*_x_*_1_(0) and *R_x_*_2_*_x_*_2_(0) are the autocorrelation functions at Positions 1 and 2 respectively when *τ* = 0. The cross-correlation function *R_x_*_1_*_x_*_2_(*τ*), is related to the Fourier Transform of the cross-spectral density (CSD) function *S_x_*_1_*_x_*_2_(*ω*) by [16]:

(4) $$R_{x_{1}x_{2}}(\tau )=\frac{1}{2\pi }\int_{-\infty }^{+\infty }S_{x_{1}x_{2}}\left(\omega \right)e^{\text{i}\omega \tau }d\omega$$

where $\text{i}=\sqrt{-1}$. Note that *S_x_*_1_*_x_*_2_(*ω*) can be written as |*S_x_*_1_*_x_*_2_(*ω*)|*^e^*^i^*^φ^*^(*ω*)^ in which |*S_x_*_1_*_x_*_2_(*ω*)| is the modulus and *φ*(*ω*) is the phase between the two signals at frequency *ω*. It was shown in [13] that the time delay can be determined approximately from the phase spectrum within a bandwidth of *m* discrete frequencies. It is given by:

(5) $$T_{0}\approx -\frac{\overset{m}{\underset{j=1}{\text{∑}}}\left|S_{x_{1}x_{2}}\left(\omega_{j}\right)\right|\phi \left(\omega_{j}\right)\omega_{j}}{\overset{m}{\underset{j=1}{\text{∑}}}\left|S_{x_{1}x_{2}}\left(\omega_{j}\right)\right|\omega_{j}^{2}}$$

Note that Equation (5) is exact only if the phase is linear (pure delay) without any significant distortion, e.g., distortions due to the dynamics of the system, and if the phase passes through the origin (*i.e.*, *φ* = 0 at *ω* = 0). Otherwise it provides a least-square best-fit of the time delay estimate. Moreover, the correct choice of the frequency bandwidth over which the calculation is performed is essential for the accuracy of this estimate [12,13]. Given these conditions it is possible to determine the time delay from the gradient of a straight line fit to the phase spectrum weighted by the modulus of the cross-spectrum at each frequency. This demonstrates the importance of the modulus of the cross-spectrum, as well as the phase in the calculation of the time delay.

## 3. Filtering Effect of the Pipe and Sensors

In this section, the simple model of the pipe-sensor system proposed in [11], is briefly reviewed with specific focus on the plastic pipe system used in the experimental work reported in Section 4. This model is extremely simple, but it is believed that it captures the main dynamic effects of the pipe and the sensor, which determine the bandwidth over which leak noise can be measured in practice, and the shape of the cross-correlation function. An infinite pipe is assumed, so that there are no wave reflections at pipe discontinuities. Furthermore, basic models of the transducer response are assumed (*i.e.*, dynamics due to internal resonances in the transducers are neglected).

As the noise propagates through the pipe, the high frequencies are attenuated because of damping in the pipe-wall and radiation of noise into the surrounding medium. Moreover the signals are further filtered by the sensors. The combined effect of the pipe and the sensors can be described by the frequency response function (FRF) between the acoustic pressure at the leak location and the sensor output (pressure, velocity or acceleration). For the sensor at Position 1 this is given by [11]:

(6) $$H(\omega ,d_{1})={\left(\text{i}\omega \right)}^{n}A_{n}e^{-\omega \beta d_{1}}e^{-\text{i}\omega d_{1}/c}$$

where *β* is the attenuation factor, *n* is related to the type of sensor being used and *A_n_* is a gain related to the pipe and sensor. For a hydrophone, *n* = 0 so that *A*_0_ = *A*_hyd_ (no units) where *A*_hyd_, is the gain of the hydrophone measurement system. For a geophone placed on the pipe-wall, *n* = 1 so that *A*_1_ = *A*_geo_ *a***^2^**/(*Eh*) (m**^3^**/N) where *A*_geo_ is the gain of the geophone measurement system. For an accelerometer placed on the pipe-wall, *n* = 2 so that *A*_2_ = *A*_acc_ *a***^2^**/(*Eh*) where *A*_acc_ is the gain of the accelerometer measurement system. At low frequencies, which is the case for leak detection in plastic pipes, the noise radiation into the surrounding medium can be neglected so that the attenuation factor is simply related to the loss in the pipe-wall and is given by [6]:

(7) $$\beta =\frac{1}{c_{f}}\frac{\eta Ba/Eh}{{\left[1+(2Ba/Eh)\right]}^{1/2}}$$

where *η* is the damping in within the pipe wall, *c_f_* and *B* are the free-field fluid wavespeed and the fluid bulk modulus of elasticity, respectively, and *E*, *a* and *h* are the Young's modulus of the pipe wall, the mean pipe radius, and the pipe wall thickness, respectively. The speed of leak noise propagation in the pipe (coupled motion of the fluid and in the pipe wall) is given by [6]:

(8) $$c=\frac{c_{f}}{{\left(1+2Ba/Eh\right)}^{\frac{1}{2}}}$$

**Figure 2.**
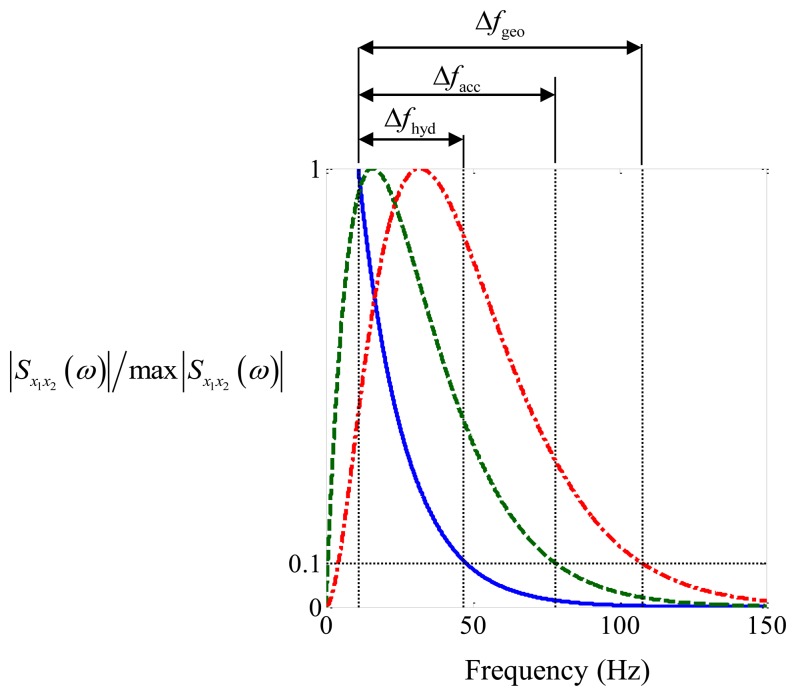
|*S_x_*_1_*_x_*_2_(*ω*)|/max|*S_x_*_1_*_x_*_2_(*ω*)| for different type of sensors. Solid blue line, hydrophone; dashed green line, geophone; dotted-dashed red line, accelerometer. max|*S_x_*_1_*_x_*_2_(*ω*)| is the maximum value of |*S_x_*_1_*_x_*_2_(*ω*)| within the frequency range greater than or equal to 10 Hz.

Now, the cross-spectrum between the two sensor outputs, each fitted either side of a leak, is given by [9]:

(9) $$S_{x_{1}x_{2}}\left(\omega \right)=S_{ll}\left(\omega \right)\left|H^{*}\left(\omega ,d_{1}\right)H\left(\omega ,d_{2}\right)\right|e^{\text{i}\omega T_{0}}$$

where *s_ll_*(*ω*) is the power spectral density of the acoustic pressure due to the leak at the leak location. It is clear from Examining Equations (5) and (9) it can be seen that |H*(*ω,d*_1_) *H* (*ω,d*_2_)| is an important factor in the estimation of the time delay. Using the properties in Table 1 for the high performance polyethylene (HPPE) pipe used in the experiments described in the next section, and assuming that *S_ll_* (*ω*) is constant within the frequency range of interest [9], |*S_x_*_1_*_x_*_2_(*ω*)|/max|*S_x_*_1_*_x_*_2_(*ω*)| is plotted for a pipe with hydrophones, geophones and accelerometers in Figure 2.

Note that max|*S_x_*_1_*_x_*_2_(*ω*)| is the maximum value of |*S_x_*_1_*_x_*_2_(*ω*)| within the frequency range greater than or equal to 10 Hz. This particular frequency was chosen because in practice a high-pass filter has to be used to remove low frequency background noise, and it has been found in practice that below about 10 Hz the measured signals are dominated by background noise.

It can be seen from Figure 2 that the pipe together with the hydrophones simply acts as a low-pass filter. The peak occurs at 10 Hz (as it is set to zero below this frequency because of background noise, as mentioned above) and is one tenth of this value at a frequency of about 47 Hz. For both the geophones and the accelerometers, the combined pipe-sensor system acts as a band-pass filter with peaks occurring at about 16 Hz and 32 Hz, respectively. The normalized CSDs are one tenth of the peak values at frequencies of about 78 Hz and 108 Hz, respectively.

Filtering the signals creates additional peaks in the cross-correlation function, which can mask the main peak due to the time delay. A measure of the height of these peaks compared to the main peak is the ratio Δ*f*/*f_c_* [12], where Δ*f* = *f*_upper_ − *f*_lower_ in which *f*_upper_ is the upper cut-off frequency and *f*_lower_ is the lower cut-off frequency of the band-pass filter, and *f_c_* = (*f*_upper_ + *f*_lower_)/2 is the central frequency of the band-pass filter. A value close to 2 (which is the maximum value that Δ*f*/*f_c_* can have) indicates that the additional peaks are small compared to the main peak [12]. Using 10 Hz as the lower frequency, and (arbitrarily) choosing the upper frequency to coincide with the values of |*S_x_*_1_*_x_*_2_(*ω*)|/max|*S_x_*_1_*_x_*_2_(*ω*)| = 0.1 results in the values given in Table 3 below, along with the assumed values of *f*_lower_ and *f*_upper_. It can be seen that for the particular configuration of interest here, the best sensor to use based on this criterion should be an accelerometer. There are, of course, other criteria, and these will be discussed later in the paper.

## 4. Experiment

### 4.1. Experimental Procedure

**Figure 3.**
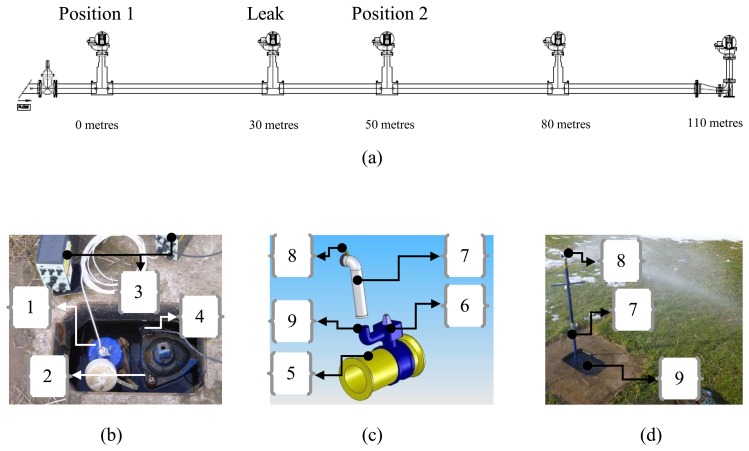
Details of the Blithfield pipe test-rig. (**a**) schematic showing the distances and the excitation/measurement positions and the leak position; (**b**) One of the access points and part of the instrumentation used. {1} Hydrophone; {2} Accelerometer; {3} Charge amplifier; {4} Geophone; (**c**) Sketch of the device used for generating the leak condition. {5} Water distribution plastic pipe; {6} Main valve; {7} Standpipe; {8} Secondary valve; {9} Hydrant; (**d**) Photograph showing the leak from the standpipe.

The experiments were conducted on a 110 m long bespoke pipe rig located at Blithfield reservoir in Staffordshire, UK. Some details of the pipe rig are shown in Figure 3, the properties of which are given in Table 1. Figure 3a shows a schematic of the pipe rig, in which the position of the leak and the two sensors can be seen. The end of the pipe close to Position 1 is connected to the mains water distribution pipe, which supplies water at a pressure of about 6 bar. The access points are set in concrete to provide a rigid support for the pipe connections, while the pipe sections are buried in the ground at a depth of about 0.8 m. Three sensors (hydrophone, geophone and accelerometer) were positioned at the access points at Positions 1 and 2 which were 30 m and 20 m from the leak respectively. A photograph of one these positions is shown in Figure 3b. Figure 3c shows a schematic of the main valve, which is at each at access point, and the standpipe which was fitted to the access point where the leak was induced.

**Figure 4.**
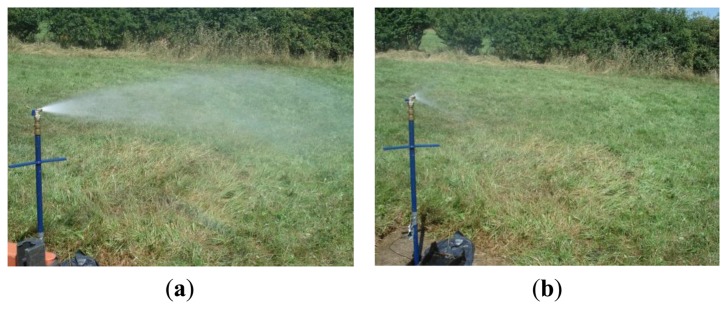
Leak induced in the pipe. (**a**) Strong leak; (**b**) Weak leak.

The leak was induced by opening the secondary valve and can be seen in Figure 3d. Although this is not a sub-surface leak it does generate a noise which is very similar to a leak below the surface, as the mechanism for leak noise is the turbulent nature of the fluid as it passes through an orifice or a leak [17,18]. In this work two different leak strengths were induced; a strong leak by opening the secondary valve fully, and a weak leak by partially closing this valve. These conditions can be seen in the photographs in Figures 4.

The leak noise sensed by the three transducers at Positions 1 and 2 was measured simultaneously for one minute using DATS [19]. The sampling frequency was set to 5 kHz, and a frequency resolution of 1 Hz was used in the subsequent spectral analysis. The details of the transducers and part of the instrumentation used in the experiment are given in Table 2. Analysis of the data collected is described in the next Section.

### 4.2. Data Processing

Prior to analysis, the data from all the sensors were passed through band-pass filters with lower and upper limits set to 10 Hz and 150 Hz, respectively. As mentioned previously, a frequency of 10 Hz was chosen for the lower limit to remove background noise due to the environment. The upper frequency of 150 Hz was chosen based on an estimate of the attenuation of the leak noise signal at the frequency. The attenuation in dB/m at frequency *ω* is given by 8.67*βω* [9]. Combining this with Equation (7) results in an attenuation of about 1.6 dB/m at 150 Hz. Thus, at 20 m and 30 m from the leak, the attenuation in the leak noise will be about 32 dB and 48 dB at 150 Hz, respectively. It was expected, therefore, that the signal to noise ratio at frequencies greater than 150 Hz would be very small.

It was found that one of the signals from a geophone had a large 50 Hz component from the mains electrical supply that could not be removed in the field, so each geophone signal was also subsequently passed through a set of notch filters set at 50 Hz, 100 Hz and 150 Hz.

**Figure 5.**
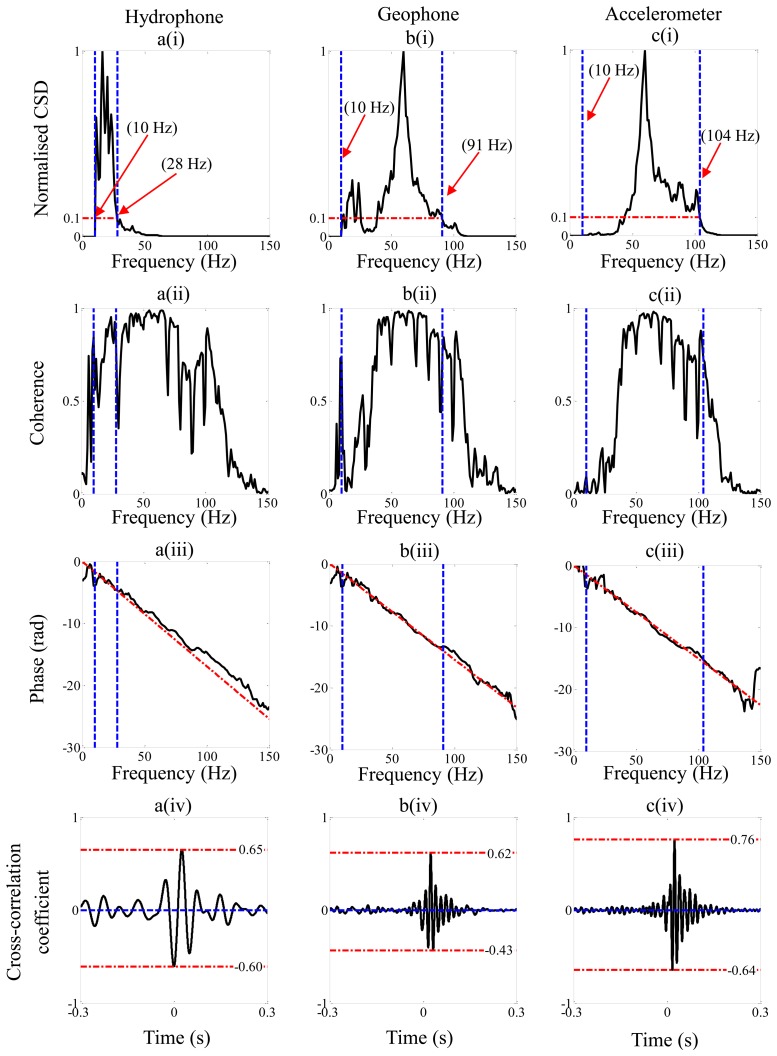
Analysis of the strong leak data from the test-rig. (**a**) Hydrophone; (**b**) Geophone; (**c**) Accelerometer. (i), Normalized CSD with respect to the maximum amplitude between 10 Hz and 150 Hz. (ii) Coherence. (iii) Phase. (iv) Cross correlation coefficient.

Figure 5 shows the processed data from the strong leak measured using the hydrophones, geophones and accelerometers respectively. The subplots (i), (ii) and (iii) correspond to frequency domain representations, namely the normalized modulus of the CSD with respect to the maximum value between 10 Hz and 150 Hz, the coherence and the phase, respectively. The subplot (iv) corresponds to the cross-correlation coefficient, from which the time delay is estimated. The key parameters extracted from the data are given in Table 3, so they can be compared directly with those predicted using the simple model.

Examining the normalized CSD in Figures 5a(i), 5b(i) and 5c(i), it can be seen that the frequency range with a lower frequency of 10 Hz and an upper frequency given by when the normalized CSD is equal to 0.1, is small (10 Hz–28 Hz) for the hydrophone measured data compared to the geophone (10 Hz–91 Hz) and accelerometer (10 Hz–104 Hz) measured data. These frequency ranges are marked on the figures for clarity. As discussed in Section 3 this band-pass filtering feature is partly due to the combined effects of the pipe and the sensors.

By examining Figures 5a(ii), 5b(ii) and 5c(ii), it can be seen that there is reasonably good coherence in all three cases over a wide frequency range. The unwrapped phase spectra are shown in Figures 5a(iii), 5b(iii) and 5c(iii). It can be seen that although there is approximately straight line behavior over a relatively wide frequency range, there are some deviations from this behavior. These could be due to reflections at low frequencies due to discontinuities in the pipe [14], and the dynamic behavior of the system at higher frequencies [12]. As discussed in Section 2, the time delay is given by the slope of the phase gradient given by a weighted least squares fit to the phase (also shown in the figures), where the weighting factor is the modulus of the cross-spectrum between the measured signals. This is clearly affected by the type of sensor used as seen in Figures 5a(i), 5b(i) and 5c(i), which determines the effective bandwidth of the data used in the time delay calculation of each case. The cross-correlation coefficients calculated from the time series from the three types of sensors are shown in Figures 5a(iv), 5b(iv) and 5c(iv). The time delays corresponding to the peaks in these graphs are 25.8 ms for hydrophone measured data, 24.4 ms for geophone measured data and 24 ms for accelerometer measured data. Using these data together with Equation (1) and assuming a wavespeed of 356 m/s given in Table 1 results in an estimated distance *d*_2_ of 20.41 m, 20.66 m and 20.72 m for hydrophone, geophone and accelerometer measured data respectively. These data are also given in Table 3 for ease of reference.

**Figure 6.**
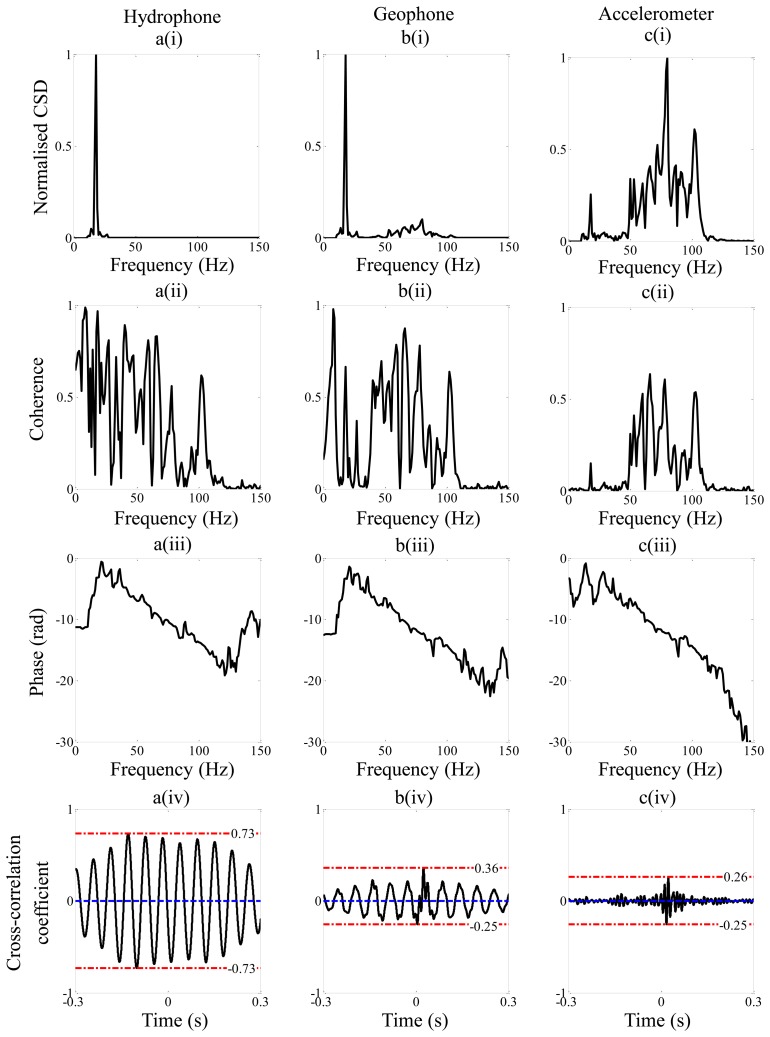
Analysis of the weak leak data from the test-rig. (**a**) Hydrophone; (**b**) Geophone; (**c**) Accelerometer. (i), Normalized CSD with respect to the maximum amplitude between 10 Hz and 150 Hz. (ii) Coherence. (iii) Phase. (iv) Cross correlation coefficient.

Figure 6 shows the processed data corresponding to the weak leak. Examining the normalized CSD in Figures 6a(i), 6b(i) and 6c(i), it can be seen that much energy is contained in a narrow band of frequencies between 18 Hz and 22 Hz, especially in the hydrophone and geophone data. This was not due to the leak, but was thought to be related to water flow in the mains pipe which fed the test rig (so-called out-of-bracket noise). The reason why the accelerometer data in Figure 6c(i) was not affected as much by this noise is because of the additional high-pass filter effect due to the sensors which is predicted by the model of the pipe-sensor system (see Figure 2).

By comparing the coherence in the weak and the strong leak cases it can be seen that the coherence was much reduced in the weak leak case resulting in a much lower signal to noise ratio, which is to be expected. In the unwrapped phase spectra for the hydrophone and the geophone a phase with positive gradient can be seen at low frequencies. This is related to the out-of-bracket noise rather than the leak noise. The cross-correlation coefficients are shown in Figures 6a(iv), 6b(iv) and 6c(iv). It is evident that only the geophone and the accelerometer are effective at detecting the leak in this case, with peaks occurring at 24.8 ms and 24 ms respectively. The quality of the correlation is much diminished, however, compared to the strong leak case.

## 5. Discussion

The key parameters extracted from the simple model and the experimental test for the strong leak are summarized in Table 3. The time delays are extracted directly from the experimental data and these need to be processed to give the leak position. To do this the speed at which the noise propagates needs to be known or estimated. This could be estimated from measurements using the three different sensors, but different results would be obtained in each case [12], as mentioned previously in Section 2. Thus, to make a meaningful comparison between the three sensors, the time delay estimates should be considered. They cannot, however, be compared against a reference value as this is unknown (and would be unknown for any practical case). To simplify the comparison, a nominal wave speed is calculated using Equation (8) (given in Table 1), so that the distance from Position 2 can be calculated using Equation (1). Of course this cannot be taken as a true estimate of the position of the leak but it does facilitate a comparison between the sensors (and the wave speed is mainly estimated this way in practice).

**Figure 7.**
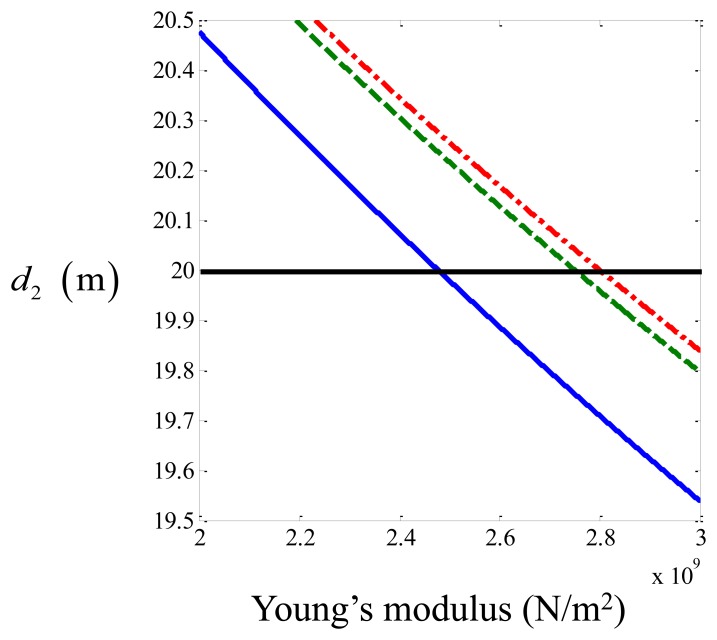
Estimate of the leak position as a function of the Young's modulus of the pipe calculated using different types of sensors. solid blue line, hydrophone; dotted green line, geophone; dashed red line, accelerometer.

It can be seen in Table 3 that the distance estimated using the three different sensor types varies by less than 2%. This is to be expected, because for a plastic pipe with dimensions of those in the test rig, the acoustic behavior of the fluid is well-coupled to the vibration of the pipe [6]. Although it appears that the hydrophone data gives the most accurate estimate, this will change if the speed of noise propagation changes. It can be seen from Equation (8) that the speed of noise propagation is dependent on the Young's modulus of the pipe which varies with temperature and this was not known precisely. To illustrate how sensitive the predictions are on the speed of noise propagation, Equation (1) is combined with Equation (8), and the estimated leak position is plotted as a function of the Young's modulus of the pipe. This is shown in Figure 7, using the three time delays estimated from the measured data. It can be seen that if the Young's modulus of the pipe was about 2.8 × 10**^9^** N/m**^2^** corresponding to a wave speed of about 416 m/s then the geophone and accelerometer data would give a better prediction of the location of the leak. Almeida [12], measured the wavespeed in the Blithfield pipe-rig at different periods over 3 years using accelerometers. He found that the wavespeed changes drastically from season to season, varying from 350 m/s to 420 m/s. Thus, it is not possible to say which sensor gives the most accurate prediction of leak location (this might be possible only in the most controlled laboratory conditions, which would not reflect the real situation in the field). It is possible to state, however, that all sensors were able to detect the leak and provide estimates for the leak location in situations in which the signal to noise ratio is not too low that are consistent with the uncertainty of the wave speed estimate. Moreover, the filtering effects of the pipe-sensor system are in qualitative agreement with the predictions from the simple model.

The second parameter that gives an indication of the prominence of the peak compared to minor peaks surrounding it in the cross-correlation function is the ratio Δ*f*/*f_c_* as discussed in Section 3. This is heavily dependent on the sensor type and leak strength. For strong leak data, it can be seen that, because the geophone and accelerometer amplify the high frequency components of the leak noise in the measured signals, the bandwidth is increased, and hence Δ*f*/*f_c_* is larger for these sensors compared to the hydrophone. The theoretical values of this quantity together with the experimental values from the strong leak are given in Table 3. It can be seen that for the geophone measured data, and in particular the accelerometer measured data, the theoretical and experimental values are very similar. There is a discrepancy with the hydrophone measured data, with the higher frequency components being attenuated at a much greater rate than that predicted as can be seen by comparing Figures 2 and 4a(i), thus limiting the effective bandwidth of this data. The reason for this is uncertain, but could be due to additional acoustic behavior of the system which was not captured by the simple analytical model. Thus, in the particular situation where the leak is considered strong, the most effective sensors for use in leak detection would be either geophones or accelerometers. For the weak leak case considered in this paper, the ratio Δ*f*/*f_c_* for the hydrophone and geophone are significantly reduced when compared with the strong leak case. This can be seen by comparing the values for the weak leak given in Table 4, with those for the strong leak given in Table 3. As mentioned in Section 3, the value of Δ*f*/*f_c_* is measure of the shape of the cross-correlation function and hence the prominence of the peak in this function which is related to the leak. It is clear that the peak is not discernible in the hydrophone-measured data, and it is much diminished in the geophone-measured for the weak leak compared to the strong leak.

In some situations, the structural sensors, accelerometers and geophones can pick up a significant of low frequency background noise and thus it is not possible for them to sense low frequency leak noise. This becomes particularly troublesome when the attenuation factor is large or the distance between the sensors and the leak is large as the high frequencies are heavily attenuated resulting in a very small frequency range where there is leak noise in the measured data. As can be seen in Equation (7) the attenuation factor is dependent upon the damping in the pipe wall which is governed by the pipe material, and the size and thickness of the pipe. Thus, a larger diameter pipe has a higher attenuation factor than a smaller diameter pipe if the pipe material remains the same. In some situations, therefore, it is desirable to use hydrophones as they are more sensitive to low frequencies compared to the structural sensors considered. It should be noted, however, that hydrophones have the disadvantage of being invasive.

## 6. Conclusions

This paper has described an experimental study to investigate the combined filtering effects of the sensors and pipe on a bespoke plastic water pipe test-rig for the purposes of leak detection. The sensors considered were hydrophones, geophones and accelerometers. Two different leaks were considered; a strong leak where the signal to ratio was high, and a much weaker leak strength where this was not the case. It was found that the experimental results for a strong leak were broadly in agreement with those predicted from a simple analytical model proposed in [10,11]. The main features are that the hydrophone-pipe system is more sensitive at low frequencies with the pipe acting as a low-pass filter, and the geophone and the accelerometer combined with the pipe effect results in band-pass filter behavior for these systems.

For the strong leak all three sensors were capable of detecting and locating the leak. However the weak leak could not be detected by the hydrophone sensors, as there was noise in a narrow range of low frequencies that were not related to the leak and dominated the signals from these sensors. This also affected the geophone data to some extent and the accelerometer data much less so, which meant that only the accelerometer could clearly detect and locate the leak in this case. The band-pass filter effect of the pipe and these sensors is the reason why this occurred.

A quality measure for the data was also presented, which is the ratio of the bandwidth over which the analysis is carried out divided by the centre frequency of this bandwidth. Based on this metric, the accelerometer was found to be the best sensor to use for the test rig described in this paper. However, if the attenuation factor in the pipe is large, either because of damping or because of pipe geometry, then it may be desirable to use hydrophones even though they are invasive sensors. It was also demonstrated that accurate location of the leak position is heavily dependent upon the precision with which the leak noise propagation speed is known, and for a plastic pipe, this is independent of the type of sensor being used.

## Figures and Tables

**Table 1.** Properties of the experimental test-rig.

Mean radius of the pipe	75 mm
Pipe-wall thickness	9.85 mm
Young's modulus	2 × 10**^9^** N/m**^2^**
Loss factor of the pipe	0.1
Bulk modulus of water	2.2 × 10**^9^** N/m**^2^**
Free-field wavespeed in water	1,500 m/s
Attenuation factor, *β*	1.99 × 10**^−4^**N/m**^2^**
Wave speed of leak noise, *c*	356 m/s

**Table 2.** Instrumentation used in the experiment.

**Device**	**Manufacturer**	**Type**
Hydrophones	Bruel and Kjaer	8103
Geophones	Ion	SM-24
Accelerometers	Bruel and Kjaer	4383 and 4384
Charge Amplifiers	Bruel and Kjaer	2635
Acquisition System	Prosig	DATS

**Table 3.** Key parameters determined from the leak data (theory and experiment (strong leak)).

**Sensor Type**	**Theory**				**Experiment**			
	*f*_lower_ (Hz)	*f*_upper_ (Hz)	$\frac{\Delta f}{f_{c}}$	*d*_2_ (m)	*f*_lower_ (Hz)	*f*_upper_ (Hz)	$\frac{\Delta f}{f_{c}}$	*d*_2_ (m)
Hydrophone	10	47	1.30	20	10	28	0.95	20.41
Geophone	10	78	1.55	20	10	91	1.6	20.66
Accelerometer	10	108	1.66	20	10	104	1.65	20.72

**Table 4.** Key parameters determined from the weak leak data (experiment only).

**Sensor Type**	**Experiment**			
	*f*_lower_ (Hz)	*f*_upper_ (Hz)	$\frac{\Delta f}{f_{c}}$	*d_2_* (m)
Hydrophone	10	19	0.62	-
Geophone	10	19	0.62	20.58
Accelerometer	10	87	1.59	20.73

## References

[1] McMahon W., Burtwell M.H., Evans M. (2005). Minimising Street Works Disruption: The Real Costs of Street Works to the Utility Industry and Society.

[2] Muggleton J.M., Brennan M.J. (2012). The use of acoustics in the water industry. Water Sewerage J..

[3] Muggleton J.M., Brennan M.J., Gao Y. (2011). Determing the location of buried plastic water pipes from measurements of ground surface vibration. J. Appl. Geophys.

[4] Romanova A., Bin Ali M.T., Horoshenkov K.V. (2011). A non-intrusive acoustic method of sewer pipe survey—A novel, inexpensive and rapid primary survey technique. AWA Water J. Asset Manag..

[5] Fuchs H.V., Riehle R. (1991). Ten years of experience with leak detection by acoustic signals analysis. Appl. Acoust..

[6] Muggleton J.M., Brennan M.J., Pinnington R.J. (2002). Wavenumber prediction of waves in buried pipes for water leak detection. J. Sound Vib..

[7] Muggleton J.M., Brennan M.J., Linford P.W. (2004). Axisymmetric wave propagation in fluid-filled pipes: Wavenumber measurements *in-vacuo* and buried pipes. J. Sound Vib..

[8] Hunaidi O., Chu W.T. (1999). Acoustical characteristics of leak signals in plastic water distribution pipes. Appl. Acoust..

[9] Gao Y., Brennan M.J., Joseph P.F., Muggleton J.M., Hunaidi O. (2004). A model of the correlation function of leak noise in buried plastic pipes. J. Sound Vib..

[10] Muggleton J.M., Brennan M.J. (2005). Axisymmetric wave propagation in buried, fluid-filled pipes: Effects of the wall discontinuities. J. Sound Vib..

[11] Gao Y., Brennan M.J., Joseph P.F., Muggleton J.M., Hunaidi O. (2005). On the selection of acoustic/vibration sensors for leak detection in plastic water pipes. J. Sound Vib..

[12] Almeida F.C.L. (2013). Improved Acoustic Methods for Leak Detection in Buried Plastic Water Distribution Pipes. Ph.D. Thesis.

[13] Brennan M.J., Gao Y., Joseph P.F. (2007). On the relationship between time and frequency domain methods in time delay estimation for leak detection in water distribution pipes. J. Sound Vib..

[14] Brennan M.J., Joseph P.F., Gao Y. (2009). On the effects of reflections on time delay estimation for leak detection in buried plastic water pipes. J. Sound Vib..

[15] Bendat J.S., Piersol A.G. (2000). Random Data: Analysis and Measurement Procedures.

[16] Knapp C.H., Carter G.C. (1976). The generalized correlation method for estimation of time delay. J. Sound Vib..

[17] Papastefanou A.S., Joseph P.F., Brennan M.J. (2012). Experimental investigation into the characteristics of in-pipe leak noise in plastic water filled pipes. Acta. Acust. United Acust..

[18] Ben-Mansour R., Habib M.A., Khalifa A., Youcef-Toumi K., Chatzigeorgiou D. (2012). Computational fluid dynamic simulation of small leaks in water pipelines for direct leak pressure transduction. Comput. Fluid..

[19] Prosig Webpage. http://www.prosig.com/noise-vibration-measurement-systems/dats/.

